# Mixed-species interactions constrain diversification and shape biofilm evolution

**DOI:** 10.1093/ismeco/ycag237

**Published:** 2026-08-20

**Authors:** Sanaa Harrass, Joyce To, Parisa Noorian, Gustavo Espinoza-Vergara, Vaughn S Cooper, Scott A Rice, Diane McDougald, M Mozammel Hoque

**Affiliations:** Australian Institute for Microbiology & Infection, University of Technology Sydney, Sydney, New South Wales 2007, Australia; Australian Institute for Microbiology & Infection, University of Technology Sydney, Sydney, New South Wales 2007, Australia; Australian Institute for Microbiology & Infection, University of Technology Sydney, Sydney, New South Wales 2007, Australia; Australian Institute for Microbiology & Infection, University of Technology Sydney, Sydney, New South Wales 2007, Australia; Center for Evolutionary Biology and Medicine, University of Pittsburgh, School of Medicine, Pittsburgh, PA 15261, United States; Australian Institute for Microbiology & Infection, University of Technology Sydney, Sydney, New South Wales 2007, Australia; Australian Institute for Microbiology & Infection, University of Technology Sydney, Sydney, New South Wales 2007, Australia; Australian Institute for Microbiology & Infection, University of Technology Sydney, Sydney, New South Wales 2007, Australia; School of Engineering, Faculty of Engineering and Information Sciences, University of Wollongong, Northfields Avenue, Wollongong, New South Wales 2522, Australia

**Keywords:** biofilm, LTEE, diversity, evolution, phenotypes, mutation

## Abstract

Experimental evolution provides a powerful framework for dissecting how ecological interactions shape adaptive trajectories. Here, we evolved *Klebsiella pneumoniae, Pseudomonas protegens,* and *P. aeruginosa* in single- and mixed-species biofilm communities for 24 weeks and tracked changes in population dynamics, phenotypes, and genomes. In mono-species evolution, all three species exhibited similar dynamics of adaptation, with steadily increasing biofilm-associated populations. In contrast, mixed-species communities displayed striking compositional shifts, with *P. protegens* emerging as the dominant biofilm former and *K. pneumoniae* dominating the supernatant. Phenotypic assays revealed that all three species showed enhanced biofilm formation, but this increase was consistently greater in isolates from mono-species than mixed species communities, with *P. protegens* showing the largest gains. Beyond biofilm production, biofilm-associated isolates exhibited greater phenotypic diversification than planktonic isolates, whereas mixed-species interactions constrained diversification. Whole-genome sequencing identified species-specific putative adaptations such as *csrD* in *K. pneumoniae, yfiBNR* in *P. protegens*, and *cheA* in *P. aeruginosa* that arose early, persisted, and were enriched in mixed-species isolates. Functional assays support the contribution of these mutations to enhanced biofilm formation, with *yfiBNR* mutations in *P. protegens* increasing cyclic-di-GMP production and producing a competitive advantage that recapitulated its dominance in evolved biofilms. Our findings show that biofilm evolution fosters phenotypic diversification, whereas interspecies interactions shape adaptive trajectories, with specific mutations associated with keystone drivers of ecological dynamics in multi-species communities.

## Introduction

Microbial communities rarely exist as single-species populations in nature; instead, they persist in complex assemblages where interactions among taxa profoundly shape evolutionary trajectories, ecological stability, and functional outputs [1, 2]. Biofilms are surface-associated microbial aggregates embedded in extracellular polymeric matrices and represent a dominant lifestyle for many bacteria in natural, industrial, and clinical environments [3, 4]. This lifestyle not only confers increased tolerance to environmental stresses and antimicrobials but also imposes spatial and resource constraints that intensify both competitive and cooperative interactions [5, 6]. Such interactions can influence not only community composition but also the direction and rate of adaptive evolution [7, 8].

Long-term experimental evolution (LTEE) has been instrumental in revealing fundamental principles of microbial adaptation [9, 10]. While classic LTEE studies have focused predominantly on single-species populations grown under well-mixed conditions, there is growing recognition that community context, particularly in structured habitats like biofilms can alter selective pressures and constrain or diversify evolutionary outcomes [11]. Recent work demonstrates that interspecies interactions can promote coexistence through niche partitioning, yet can also limit phenotypic diversification by restricting available adaptive pathways [12, 13]. How these forces play out over hundreds of generations in spatially structured multispecies systems remains poorly understood.

The Gram-negative bacteria *K. pneumoniae, P. protegens,* and *P. aeruginosa* together constitute a well-established mixed-species biofilm model that has been extensively characterized by our group. These species coexist in metalworking fluids, soil habitats, and the gut of *Bombyx mori* [14, 15]*,* and prior work from our laboratories has demonstrated that this three-species community displays enhanced stress resistance [6, 16], interspecies diversity [8, 17], quorum sensing–dependent species interactions [18, 19], and associational resistance to predation [20]. These species differ in metabolic capabilities, motility, and exoproduct repertoires, which can mediate competitive and cooperative behaviors [6, 21]. In mixed-species biofilms, such traits influence spatial positioning, resource capture, and community stability [8, 22]. However, whether these differences lead to predictable patterns of dominance, diversification, and genetic adaptation over evolutionary timescales remains unclear.

Here, we used a 24-week LTEE to compare evolutionary and ecological dynamics in mono-species and mixed-species biofilm communities of *K. pneumoniae, P. protegens,* and *P. aeruginosa*. By integrating temporal population quantification, high-throughput phenotyping, and whole-genome sequencing of evolved isolates, we identified contrasting trajectories of phenotypic diversification, biofilm adaptation, and mutational targets in mono versus mixed-species contexts. Our findings reveal that while biofilm-associated evolution generally increases biofilm formation capacity, interspecies interactions constrain phenotypic heterogeneity, channel adaptation toward specific genetic pathways, and drive long-term shifts in species dominance. These results highlight how a small number of keystone mutations can couple phenotypic adaptation to community-level ecological outcomes in structured multispecies environments.

## Materials and methods

### Bacterial strains and design of LTEE

The wild-type strains of eGFP-tagged *K. pneumoniae* KP-1, eCFP-tagged *P. protegens* Pf-5 (ATCC BAA-477), and mCherry-tagged *P. aeruginosa* PAO1 (ATCC BAA-47) were used to establish single-species and mixed-species communities [6, 18]. LTEE was performed using bead transfer model under biofilm-promoting conditions using sterile polystyrene beads as attachment surfaces according to Poltak and Cooper [2]. Three and six replicate lineages were maintained for mono and mixed-species communities, respectively. M9 minimal medium supplemented with casamino acid and glucose referred to here as MCG (48 mM Na_2_HPO_4_; 22 mM KH_2_PO_4_; 9 mM NaCl; 19 mM NH_4_Cl; 2 mM MgSO_4_; 0.1 mM CaCl2; 0.04% wt/vol glucose, and 0.2% w/v casamino acids) was used to conduct the LTEE. The assay was conducted in a round-bottom, borosilicate glass tube using a rotary shaker at 60 rpm at 24°C. Beads were transferred every 3 days into 2 ml of fresh medium containing new sterile beads to propagate biofilm-associated populations. Communities were evolved for 24 weeks, with sampling performed weekly. At each transfer, a single colonized bead was moved with sterile plastic loop into the new tube containing two fresh sterile beads (alternate colored) and 2 ml of fresh MCG medium; another bead was used for downstream quantification as described below sections; the previous tube (containing the residual supernatant and the remaining beads) was discarded. This single-bead transfer regime, adapted from Poltak and Cooper [2], imposed a recurrent bottleneck on the biofilm-associated population while leaving the planktonic phase free to regrow each cycle. For mono-species communities, overnight cultures were normalized to OD600 0.5 and 100 μl was inoculated into 2 ml of MCG containing two sterile beads. For mixed-species communities, each species was normalized to OD600 0.5 and combined at a 1:3:3 ratio for KP-1, PAO1, and Pf-5, respectively, prior to inoculation, giving approximately equal starting colony-forming units (CFUs) for each of the three species [6].

### Quantification of bead-associated and planktonic populations

The bead-associated and planktonic populations were quantified every week. During, serial passaging of the beads one bead was removed from each lineage into a tube containing sterile 0.9% NaCl for quantification. The tube containing the bead was sonicated in an ultrasonic water bath (Powersonic 420, Thermoline Scientific) at medium power for 1 min, followed by vortexing vigorously to dislodge biofilm-associated cells. Planktonic populations were quantified by removing 100 μl of the supernatant from each lineage. Serial dilutions were plated on CHROMagar media to quantify CFUs for each species. For the mixed-species communities, CHROMagar allowed differentiation of *K. pneumoniae* (greenish colonies) from *Pseudomonas* spp. (white colonies) based on colony morphology. *Pseudomonas aeruginosa* were quantified by incubating one CHROMagar plate at 37°C as *P. protegens* do not grow at this temperature, while *P. protegens* were quantified by using streptomycin (50 μg/ml) into the CHROMagar medium incubated at 30°C.

### Phenotypic assays

A total of 648 single isolates (bead-associated and planktonic, across four time points and lineages) were profiled for biofilm formation (Supplementary Table S1), providing biological replication across independent replicate lineages (three mono and six mixed-species) and timepoints. Each isolate was assayed in three technical replicates within a single experiment. *Pseudomonas* spp. were subjected to motility, protease activity, and pyoverdine production assay. In addition, *P. aeruginosa* was also tested for pyocyanin production. Biofilm formation was quantified using a 96-well crystal violet assay. Overnight cultures were normalized to OD_6_₀₀ = 0.05 in MCG, incubated statically at 24°C for 24 h, stained with 0.1% crystal violet, washed, and solubilized in 95% ethanol. Absorbance was measured at 590 nm. Motility assays were performed on 0.3% agar (swimming) lysogeny broth (LB) plates, inoculated centrally with 1 μl of overnight culture and incubated at 30°C for 24 h. Motility was quantified as the diameter of colony expansion. Protease activity was assessed on LB agar supplemented with 2% skim milk. Zone diameters of casein degradation were measured after 24 h at 30°C. Siderophore production was measured as pyoverdine fluorescence (excitation 400 nm, emission 460 nm) from culture supernatants normalized to OD_6_₀₀, and pyocyanin production in *P. aeruginosa* was quantified spectrophotometrically at 520 nm following separation of the supernatants.

### Sequencing and genomic analysis

Genomic DNA was extracted from all 648 phenotyped isolates using the QIAamp 96 DNA QIAcube HT Kit (Qiagen, cat. no. 51331) according to the manufacturer’s instructions. DNA concentration was measured using the Quant-iT™ PicoGreen™ dsDNA Reagent (Invitrogen). Of the 648 isolates, 588 passed the sequencing quality requirements and were subjected to downstream analyses. Whole-genome sequencing libraries were prepared according to the Hackflex method [23] and sequenced on the Illumina NovaSeq6000 platform (2 × 150 bp paired-end). Whole-population sequencing was conducted on pooled bead-associated and supernatant fractions from each lineage (three mono and six mixed-species) across four time points. Reads were quality-trimmed using TrimGalore to remove adapter contamination and low-quality bases (≤Q30) and were checked using FastQC. The sequence reads for each of the three species from whole population samples of mixed-species communities were separated using “bowtie2,” followed by “samtools.” Filtered reads were mapped to the respective curated ancestral reference genomes, and genetic variants were identified using the breseq pipeline [24]. The curated (mutated) ancestral reference genome for the three species was built using “gdtools” in the breseq pipeline using the *K. pneumoniae* KP-1 (NZ_CP012883), *P. protegens* Pf-5 (NC_004129), and *P. aeruginosa* PAO1 (NC_002516). Genetic variants in single isolates were identified using clonal mode, while whole population samples were identified with using polymorphism mode with polymorphism frequency cut off of 0.1. Whole population analyses could not be performed for *P. aeruginosa* at Weeks 8, 15, and 24, and for the *P. protegens* Week 1 bead sample, due to insufficient sequencing reads.

### Protein structural modeling

Protein structure of the CsrD (A0A3R7K3W9), YfiB (A0A2C9EG30), and CheA (G3XCT6) were obtained from AlphaFold protein structure database (https://www.alphafold.ebi.ac.uk) [25]. Structures were visualized and rendered with the MolStar on the AlphaFold website.

The amino acid residues were manually denoted and colored in pink according to the respective nsSNPs or deletions observed on CsrD, YfiB, and CheA. The Alphafold predication model color-codes different regions of the protein based on the prediction confidence score (Predicted Local Distance Difference Test: pLDDT). Dark-blue (pLDDT >90) regions indicate very high, cyan (90 > pLDDT >70) indicate high, and yellow/orange (70 > pLDDT >50) and red (pLDDT <70) indicate low confidence prediction.

### Cyclic-di-GMP quantification

Quantification of c-di-GMP levels was performed using the c-di-GMP ELISA kit by Cayman Chemical, USA (Item #501780). Briefly, bacterial strains were cultured in MCG for overnight, normalized to OD600 = 0.4 and 1 ml of the normalized culture pelleted for lysis in 80 μl of B-PER™ buffer (Thermo Fisher Scientific). Cell lysates were serially diluted for the assay according to the manufacturer’s instructions. Concentration of c-di-GMP in the samples were calculated from the standard curve, readings outside of the recommended range and inconsistent concentrations (after dilution factor adjustment) were excluded. Corresponding sample dilution factors were multiplied to obtain the final c-di-GMP concentrations in picograms per milliliter.

### Competition assays

Competition experiments were conducted in LTEE-mimicking conditions using wild-type ancestral strains or isolates carrying prevalent mutations in *csrD* (*K. pneumoniae*), *yfiBNR* (*P. protegens*), or *cheA* (*P. aeruginosa*). Equal initial densities of each competitor were inoculated into 2 ml of MCG medium containing sterile polystyrene beads. Tubes were incubated at 24°C at 60 rpm on rotary shaker. Bead-associated and planktonic fractions were sampled after 24 h. Relative abundances were determined by plating on CHROMagar and calculating the proportion of CFUs for each species as described earlier.

### Statistical analysis

Statistical analyses were performed in R v4.3. Data were log₁₀-transformed where necessary to meet normality assumptions. Biofilm formation data were analyzed using rank-transform two-way Analysis of Variance (ANOVA) with community context (Mono vs. Mixed) and time (Weeks 8, 15, 24) as fixed factors; pairwise comparisons between community types at each timepoint used Wilcoxon rank-sum tests with Benjamini–Hochberg (BH) correction. Mutation accumulation per isolate was assessed by two-way ANOVA on log(*n* + 1)-transformed values with community context and time as fixed factors. Differences in c-di-GMP levels among strain groups were assessed using a Kruskal–Wallis test, followed by pairwise Wilcoxon rank-sum tests with BH correction. Principal component analysis (PCA) was performed using the “princomp” function on phenotypic data and visualized using “factoextra” package in R. Homogeneity of multivariate dispersion across groups was tested using the “betadisper” function from the “vegan” package with 999 permutations (PERMDISP). Motility and other phenotypic assays were compared between community types using Wilcoxon rank-sum tests. Mutation frequencies over time were visualized with ggplot2. Significance thresholds were set at *P* < .05. Genotype–phenotype associations were established by linking each isolate’s biofilm formation data to its clonal-mode breseq output, with isolates categorized as mutant or nonmutant based on the presence of nonsynonymous SNPs or small indels in *csrD, yfiBNR*, and *cheA*.

## Results

### LTEE reveals contrasting population dynamics in mono and mixed-species biofilm communities

**Figure 1:**
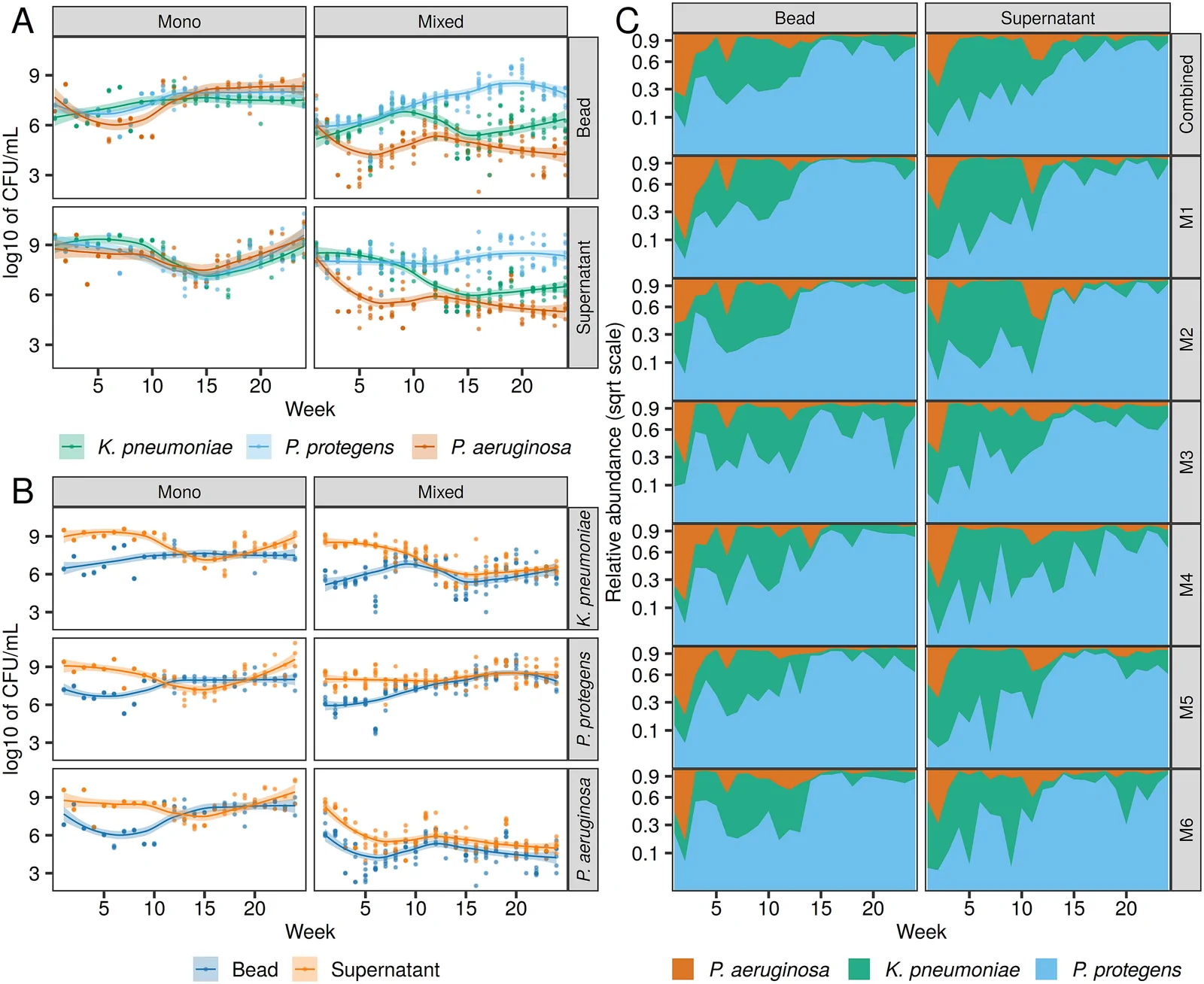
Dynamics of mono and mixed-species biofilm communities during LTEE. (A, B) Temporal changes in cell counts (log₁₀ CFU/ml) of *K. pneumoniae, P. protegens,* and *P. aeruginosa* recovered from biofilm beads and planktonic supernatants in mono and mixed-species communities. Individual points represent absolute CFU counts, while solid lines and shaded regions depict the smoothed trajectory (loess function with 95% confidence interval). (C) Relative abundance of the three species in mixed-species communities across six replicate lines (M1–M6). The first facet (top left) shows the average relative abundance across all replicates, while subsequent facets display individual community replicates. Relative abundance is plotted on a square-root-transformed scale to improve visibility of minority species.

We conducted a 24-week LTEE experiment in which *K. pneumoniae, P. protegens,* and *P. aeruginosa* were propagated either as single species or as a three-species community under repeated bead-transfer biofilm selection (Fig. 1). At each transfer, one colonized bead was transferred into fresh medium containing two new sterile beads to propagate the next culture, while a second colonized bead was removed for CFU enumeration, imposing a strong and recurrent population bottleneck while maintaining continuous opportunities for biofilm growth and dispersal. Population densities recovered from the quantification bead ranged from 10^6^ to 10^9^ CFU/ml, depending on species and time point. Using the formula *g* = log2(*N*_final_/*N*_initial_) with *N*_initial_ of ~10^7^ CFU per bead (measured directly) and *N*_final_ of ~2 × 10^9^ cells yields approximately seven to eight generations per cycle. With 56 transfers over 168 days, populations accumulated roughly 390–450 generations over the 24-week experiment.

In mono-species communities, all three species exhibited broadly similar early-phase population dynamics (Fig. 1A and B). Planktonic populations consistently exceeded bead-associated populations by approximately one to three orders of magnitude during the initial transfers, reflecting rapid regrowth in the supernatant following each bottleneck. Over successive transfers, bead-associated populations increased markedly between Weeks 1 and 24, with fold increases of ~66-, 25-, and 9-fold for *P. aeruginosa, K. pneumoniae*, and *P. protegens*, respectively. Supernatant populations increased over the same period for *P. protegens* (~12-fold) and *P. aeruginosa* (~4-fold), while *K. pneumoniae* supernatant counts declined ~3-fold. By approximately Weeks 10–12 (~100–200 generations), bead-associated and supernatant populations converged for *P. aeruginosa* and *K. pneumoniae*, consistent with preferential adaptation to the biofilm niche. The bead-to-supernatant ratio for *P. protegens* remained comparatively stable, with both fractions increasing in parallel and suggesting a broader growth advantage under LTEE conditions.

In contrast, mixed-species communities displayed strongly divergent and time-dependent population dynamics among species (Fig. 1A–C). During the initial phase of the experiment (Weeks 1–2; <50 generations), *P. aeruginosa* frequently dominated the bead-associated fraction, accounting for >50% of attached cells in several replicates (Fig. 1C). This early advantage was transient. From approximately Week 3 onward, *P. protegens* increased sharply in bead-associated abundance and rapidly became the dominant biofilm resident, persisting for the remainder of the experiment (>200 generations) across all six independent mixed-community replicates (M1–M6) (Fig. 1C). Importantly, *K. pneumoniae* and *P. aeruginosa* were not displaced; absolute CFU counts confirm that both persisted at 10^6^–10^7^ CFU/ml throughout the experiment while *P. protegens* reached 10^8^–10^9^ CFU/ml (Fig. 1A and B).

Population dynamics in the supernatant phase of mixed-species communities followed a distinct temporal pattern. *K. pneumoniae* initially dominated the planktonic fraction, consistent with rapid post-bottleneck regrowth, but this dominance declined over time, and from approximately Weeks 9–10 onward, *P. protegens* also became numerically dominant in the supernatant, frequently exceeding 80% relative abundance. The parallel rise of *P. protegens* in both fractions is consistent with its dominance on the bead seeding the subsequent supernatant at each transfer, and its sustained high planktonic abundance further suggests it was not strongly counter-selected in the planktonic phase.

Together, these results demonstrate that while single-species populations converge toward similar biofilm–planktonic equilibria over a few hundred generations, multispecies interactions fundamentally reshape evolutionary trajectories. Early transient dominance by one species giving way to stable long-term numerical dominance by another.

### Species interactions modulate the evolutionary enhancement of biofilm production

**Figure 2:**
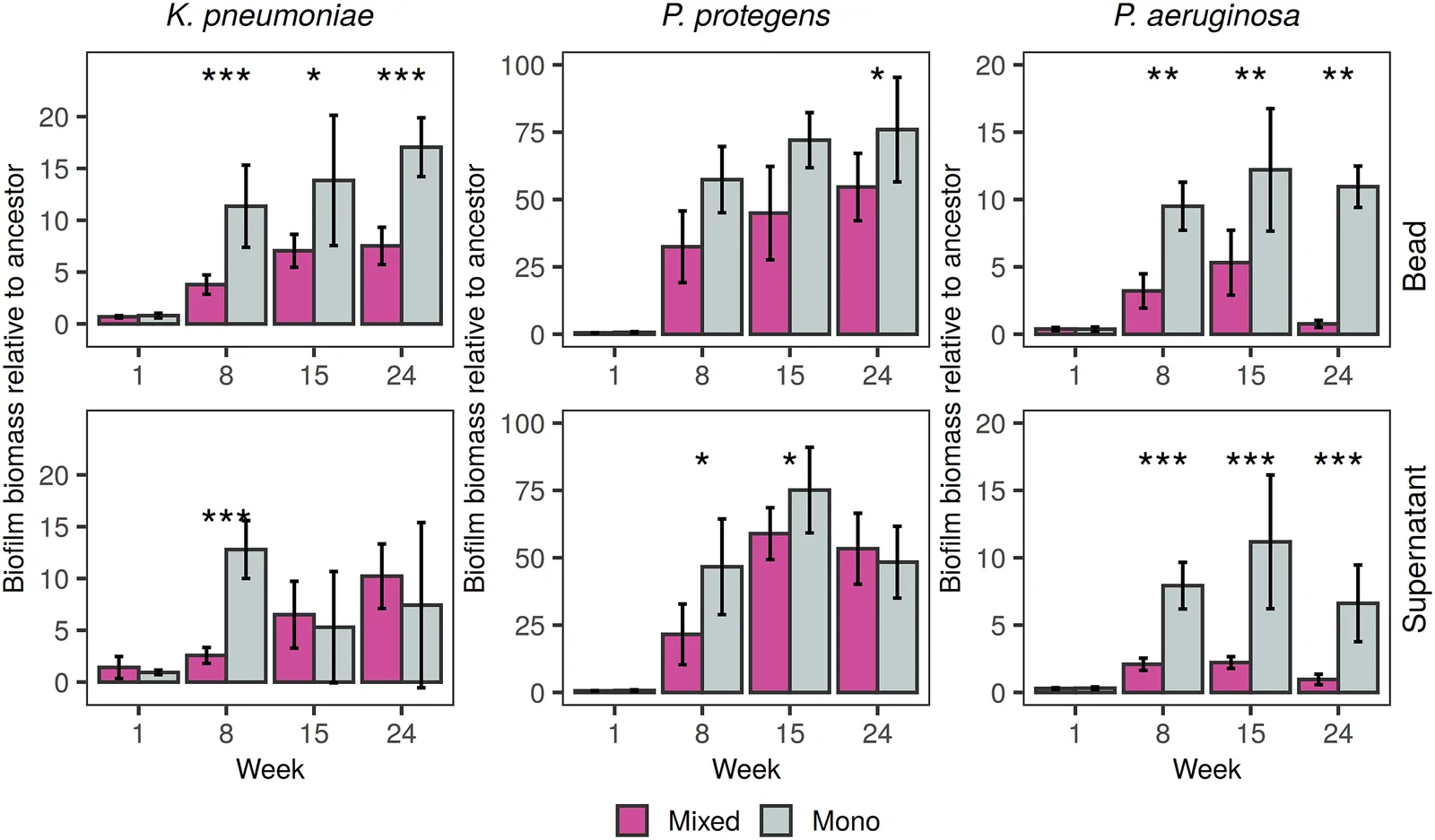
Biofilm production is constrained in mixed-species communities. The relative biofilm formation capacity compared to the respective ancestral strain are shown for the three species. Biofilm biomass was quantified using crystal violet staining assay. Data are presented as the mean of three technical replicates of respective isolates from mono and mixed communities as described in Supplementary Table S1. The error bar represents 95% CI. Statistical significance was determined using rank-transform two-way ANOVA with community context (mono vs. mixed) and time as fixed factors, followed by pairwise Wilcoxon rank-sum tests with Benjamini–Hochberg correction for multiple comparisons. Significance indicated as ^*^P < .05; ^**^P < .01; ^***^P < .001.

To investigate phenotypic changes during the LTEE, we profiled 648 single isolates collected at four time points spanning the initial, intermediate, and final stages of the experiment (Weeks 1, 8, 15, and 24; Supplementary Table S1) using various assays as described in the Materials and Methods. As expected, isolates from both mono and mixed-species communities of *K. pneumoniae, P. protegens,* and *P. aeruginosa* exhibited increased biofilm formation capacity over time (Fig. 2). At Week 1, the biofilm formation ability of all three species was comparable to their respective ancestral strains, with no significant differences observed between isolates from mono and mixed-species communities.

In Weeks 8, 15, and 24, mono isolates from beads demonstrated significantly enhanced biofilm formation compared to their mixed counterparts. Rank-transform two-way ANOVA confirmed significant community effects (Mono vs. Mixed) on bead biofilm formation in all three species (*K. pneumoniae*: *F* = 65.3, *P* < .001; *P. protegens*: *F* = 12.3, *P* < .001; *P. aeruginosa*: *F* = 64.25, *P* < .001). For *K. pneumoniae*, mono and mixed-species bead isolates produced ~16-fold and ~4-fold increased biofilm relative to the ancestor at Week 8, respectively, with pairwise differences significant at Weeks 8, 15, and 24 (all *P* < .05; Fig. 2). Among the three species, *P. protegens* exhibited the greatest capacity for biofilm production. When evolved alone on beads, *P. protegens* displayed average increases of ~57-, ~76- and ~92-fold at Weeks 8, 15, and 24, respectively. When evolved in mixed communities, isolates showed ~36-, ~53-, and ~57-fold increases, the pairwise differences between mono and mixed isolates were statistically significant at Week 24 (*P* < .05). For *P. aeruginosa*, mono bead isolates consistently exceeded mixed isolates at all three timepoints (~10-, 12-, and 11-fold vs. 6-, 5-, and 1-fold; all *P* < .01).

Overall, the biofilm assays indicated that evolution in mixed communities constrained biofilm production relative to evolution in monoculture, although both sets increased significantly. *Pseudomonas protegens* evolved to become the strongest biofilm former among the three species, and this may have accounted for its increased abundance during long-term co-evolution.

### Mixed-species interactions constrain phenotypic diversification during biofilm evolution

To investigate whether evolution in the bead biofilm model influences phenotypes beyond biofilm formation, we performed motility, protease, and pyoverdine production assays on the two *Pseudomonas* species. *P. aeruginosa* was additionally assessed for pyocyanin production. Overall, both *P. aeruginosa* and *P. protegens* exhibited reduced motility relative to their ancestral strains during LTEE (Supplementary Fig. S1). However, no consistent differences in motility emerged between mono and mixed isolates.

Protease activity in mixed isolates remained comparable to ancestral strains, whereas bead-associated isolates from monocultures frequently displayed elevated protease production (Supplementary Fig. S1). Specifically, *P. aeruginosa* bead-associated isolates from monocultures produced significantly more protease than mixed isolates at Week 24, while *P. protegens* bead-associated isolates from monocultures exceeded mixed isolates at Weeks 15 and 24. Pyoverdine production remained unchanged in *P. aeruginosa* but increased over time in *P. protegens*, with mono isolates producing more than their mixed counterparts (Supplementary Fig. S1). This enhanced siderophore production is associated with the relative increase of *P. protegens* and decline of *P. aeruginosa* abundance during LTEE, although whether iron competition is a causal driver remains to be tested experimentally. In contrast, pyocyanin production in *P. aeruginosa* steadily increased over time, with mono isolates consistently surpassing mixed isolates (Supplementary Fig. S1).

**Figure 3:**
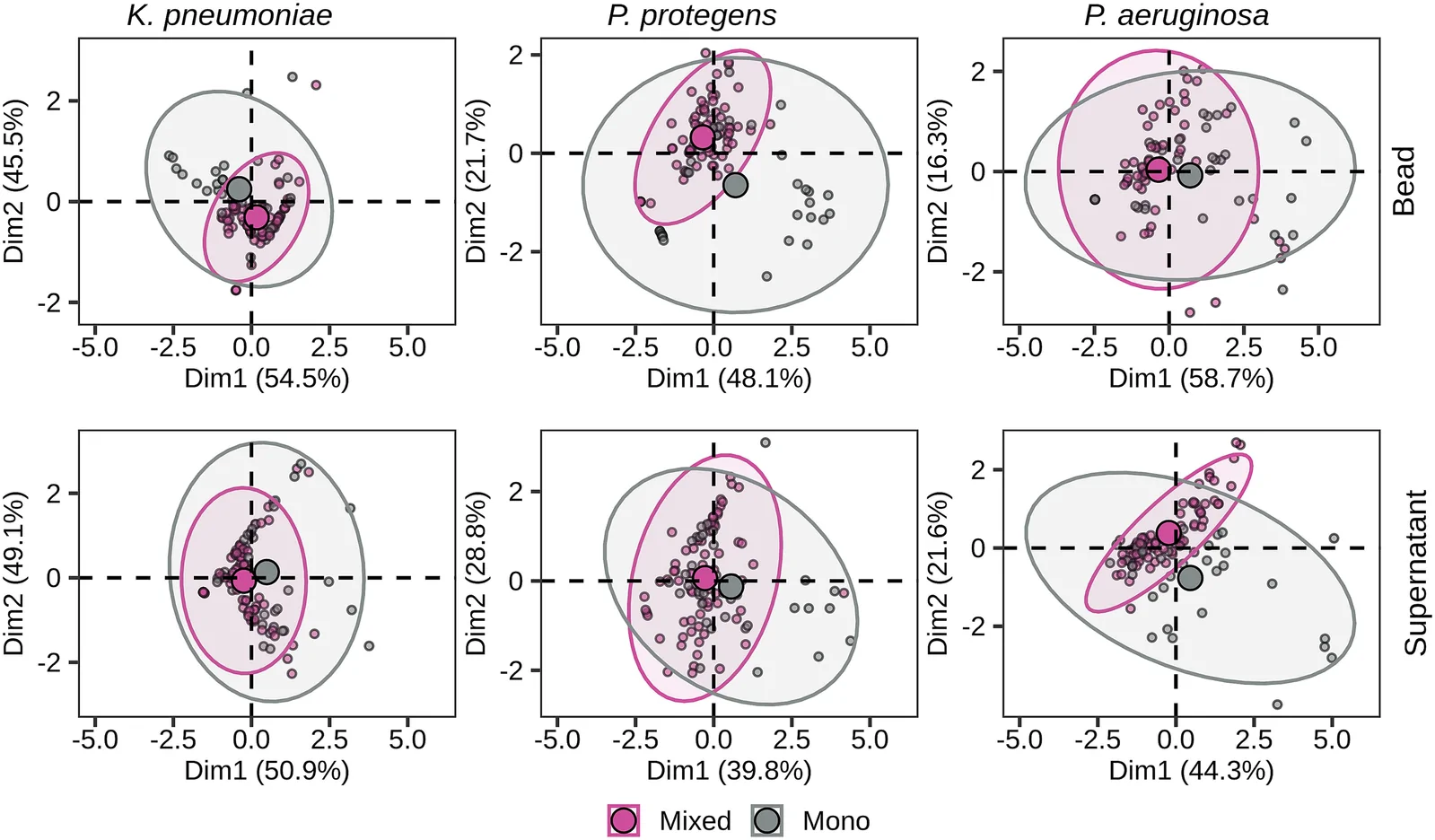
Principal component analysis (PCA) of phenotypic variation in isolates from mono and mixed-species communities. PC1 and PC2 are shown on the *x*- and *y*-axes, respectively, with variance explained indicated in brackets. The data points are grouped according to community type (mono vs. mixed), as indicated in the legend. Ellipses represent the 95% confidence interval and larger circles denote group centroids. For *K. pneumoniae*, PCA was performed on biofilm formation and OD_6_₀₀; for *P. protegens* and *P. aeruginosa*, PCA additionally included motility, protease activity, and pyoverdine production, with pyocyanin also included for *P. aeruginosa*. Multivariate dispersion was formally tested using PERMDISP (vegan::betadisper, 999 permutations) on PC1–PC2 coordinates.

To visualize overall patterns of phenotypic variation, we performed PCA using all measured traits. Across all three species, bead-associated populations showed consistently higher mean multivariate dispersion than planktonic populations (PERMDISP; all F < 1.5, *P* > .05), though these differences did not reach statistical significance (Fig. 3 and Supplementary Table S2). Similarly, isolates evolved in monoculture showed greater dispersion in PCA space than those from mixed-species communities, with this difference being statistically significant for *P. protegens* bead-associated isolates (PERMDISP; *F* = 34.3, *P* = .001) and *P. aeruginosa* planktonic isolates (*F* = 11.4, *P* = .003), and directionally consistent but nonsignificant for the remaining comparisons (Fig. 3). This contrast was most pronounced in *P. aeruginosa*, followed by *P. protegens* and *K. pneumoniae*.

These results demonstrate that biofilm-associated evolution fosters greater phenotypic heterogeneity than planktonic growth, whereas mixed-species interactions constrain diversification. Enhanced pyoverdine production in *P. protegens* and pyocyanin production in *P. aeruginosa* are associated with shifts in community composition during LTEE, though the causal contribution of these traits requires experimental validation.

### Genetic signatures of adaptation reveal species-specific evolutionary trajectories

To identify genetic changes underlying biofilm adaptation during the experiment, we sequenced 588 of the 648 phenotyped single isolates that passed library quality control at Weeks 1, 8, 15, and 24. We focused on genes showing repeated mutations from week 8 through week 24 and on genes uniquely mutated in mono versus mixed communities, and whole-population sequencing to an average of 2400× coverage. A detailed description of these analyses is provided in the Supplementary Text.

**Figure 4:**
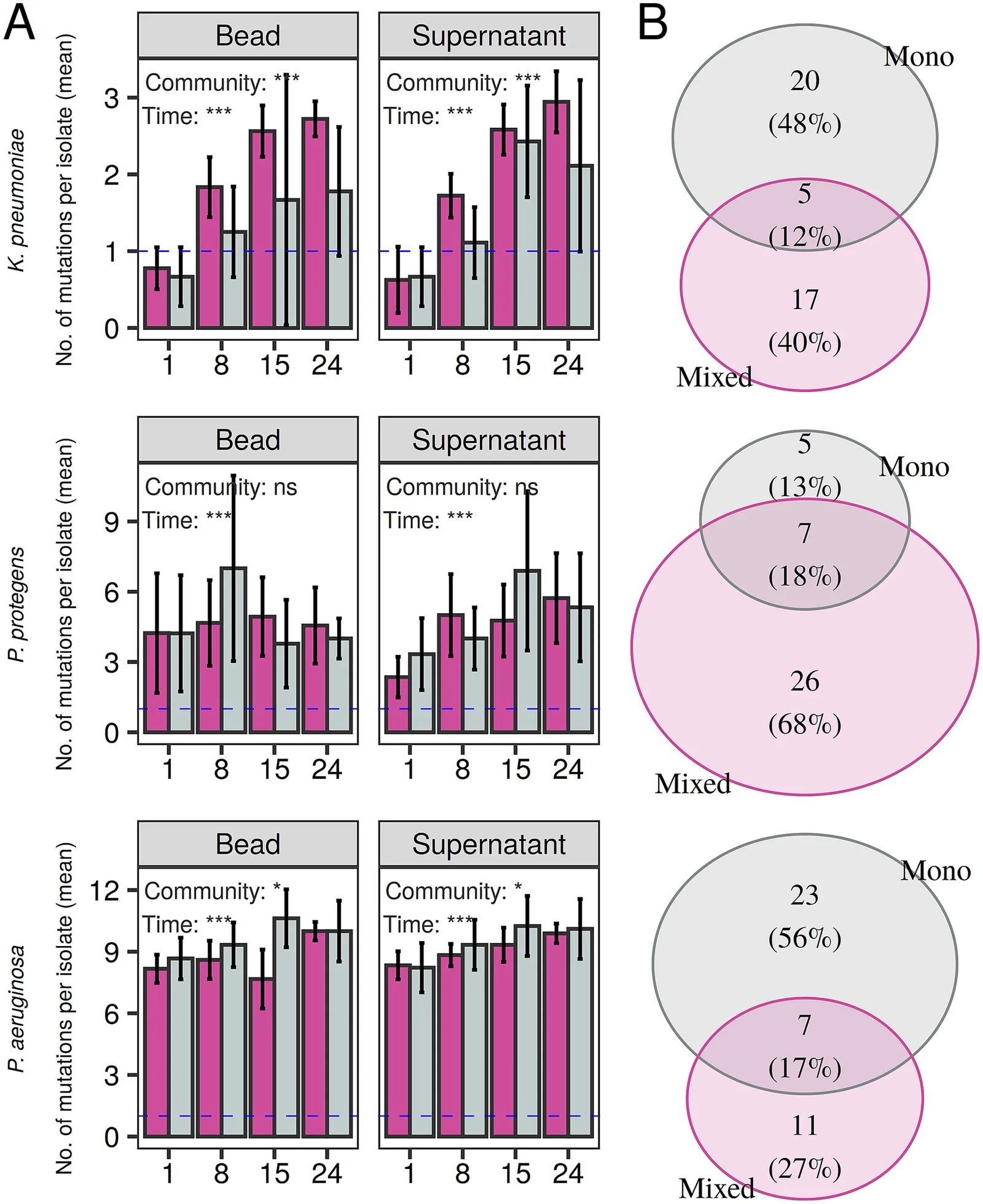
Mutational divergence and gene-level overlap between isolates from mono and mixed-species communities. (A) The mean number of mutations per isolate from mono and mixed-species communities across four time points. Data are presented as the mean and the error bar represents 95% CI. The dashed line at *y* = 1 indicates a mean mutation count of one, provided as a reference baseline. Differences in mutation accumulation between mono and mixed isolates were assessed using two-way ANOVA on log(*n* + 1)-transformed values with community context and time as fixed factors. (B) The number of shared and unique genes between mono and mixed-species isolates.

We identified 356, 617, and 691 mutations affecting 42, 38, and 41 genes in single isolates of *K. pneumoniae, P. protegens*, and *P. aeruginosa*, with isolates carrying an average of 1.8, 4.6, and 9.2 mutations, respectively (Fig. 4A). In *K. pneumoniae*, both community context and time significantly predicted mutation accumulation across bead and supernatant fractions (two-way ANOVA, *P* < .001; Fig. 4A and Supplementary Table S3), whereas *P. protegens* and *P. aeruginosa* showed weaker or fraction-specific community effects. The distribution of unique and shared mutated genes also differed among species. In *P. aeruginosa*, most mutated genes were unique to mono isolates, in *P. protegens*, most were unique to mixed isolates, and in *K. pneumoniae*, the proportions were comparable between contexts (Fig. 4B). Analysis of mutation types revealed a higher proportion of small indels from Week 8 onward in mixed-species bead isolates of *K. pneumoniae* and *P. protegens,* and in *K. pneumoniae* supernatant isolates (Supplementary Fig. S2).

**Figure 5:**
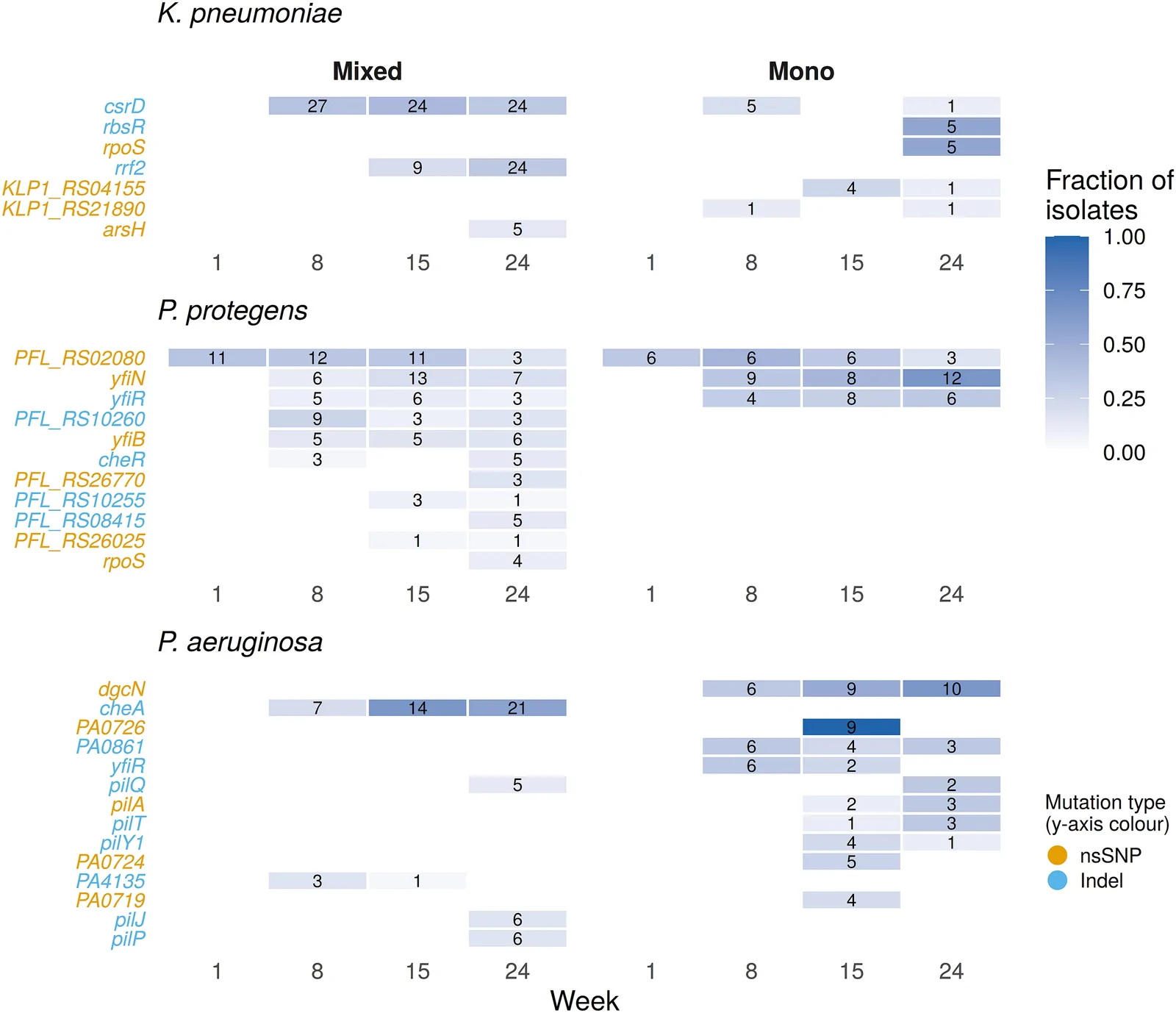
Temporal dynamics of mutations in frequently mutated genes across mono and mixed-species communities. Heatmaps showing the fraction of sequenced isolates carrying nsSNPs or small indels in the most frequently mutated genes for each species. Nonsense mutations were excluded as they were rare in *K. pneumoniae* and *P. protegens*, and in *P. aeruginosa* were overwhelmingly confined to rpoS, a general laboratory adaptation not specific to biofilm selection. Shading intensity indicates the proportion of isolates with mutations in each gene and the numbers within cells show the absolute count of mutated isolates. Only genes showing mutations in multiple independent contexts (>1 timepoint or >2 isolates) are displayed to highlight potential targets of selection. Genes are ordered by total mutation frequency across all timepoints within each species. Blank cells indicate no mutations detected.

Parallel evolution, indicated by distinct mutations in the same gene arising over time within a replicate population and independently across separate replicate populations, was most pronounced in *P. protegens* mixed communities, with seven genes showing repeated mutations, compared to two to three genes in *K. pneumoniae* and two to six genes in *P. aeruginosa* (Fig. 5). In *K. pneumoniae*, mutations in *csrD* (carbon storage regulator/RNase E specificity factor) emerged by Week 8 and persisted through Week 24, together accounting for 60%–90% of sequenced mixed isolates (Fig. 5). Whole-population sequencing confirmed a steady increase in *csrD* mutation frequency over time, reaching 25%, 50%, and 75% at Weeks 8, 15, and 24, respectively (Supplementary Fig. S3). An in-depth analysis identified a dominant nSNP at position 938 (A → G) in *csrD* (Supplementary Fig. S4). In *P. protegens*, mutations in the *yfiBNR* operon (*yfiB, yfiN, yfiR*), a tripartite module controlling intracellular c-di-GMP levels, occurred from Weeks 8 to 24, with *yfiB* mutations found exclusively in mixed isolates (Fig. 5) [26]. Whole-population data mirrored these patterns, with *yfiB* mutated only in mixed populations, while *yfiN* and *yfiR* mutations were distributed across both evolutionary contexts (Supplementary Fig. S3). In *P. aeruginosa*, small indels in the chemotaxis gene *cheA* were unique to mixed isolates and persisted from Week 8 to Week 24, whereas the diguanylate cyclase *dgcN* (PA3177) was mutated only in mono isolates (Fig. 5).

Collectively, our findings identify *csrD, yfiBNR*, and *cheA* as repeatedly selected genetic targets in *K. pneumoniae, P. protegens*, and *P. aeruginosa*, respectively. The temporal dynamics of these mutations, combined with parallel evolution across independent lineages, strongly support their adaptive significance in biofilm formation. Species-specific mutation patterns, particularly the enrichment of *yfiB* and *cheA* mutations exclusively in mixed communities, suggest distinct genetic routes to biofilm adaptation shaped by interspecies interactions.

### Mutations in *csrD, yfiBNR,* and *cheA* are associated with enhanced biofilm formation

Given that mutations in *csrD, yfiBNR,* and *cheA* (PA1458) were the most prevalent in mixed isolates of *K. pneumoniae, P. protegens*, and *P. aeruginosa*, respectively, we hypothesized that these genetic changes play a major role in biofilm formation. To test this, we categorized phenotypic data based on the presence or absence of mutations in these genes.

**Figure 6:**
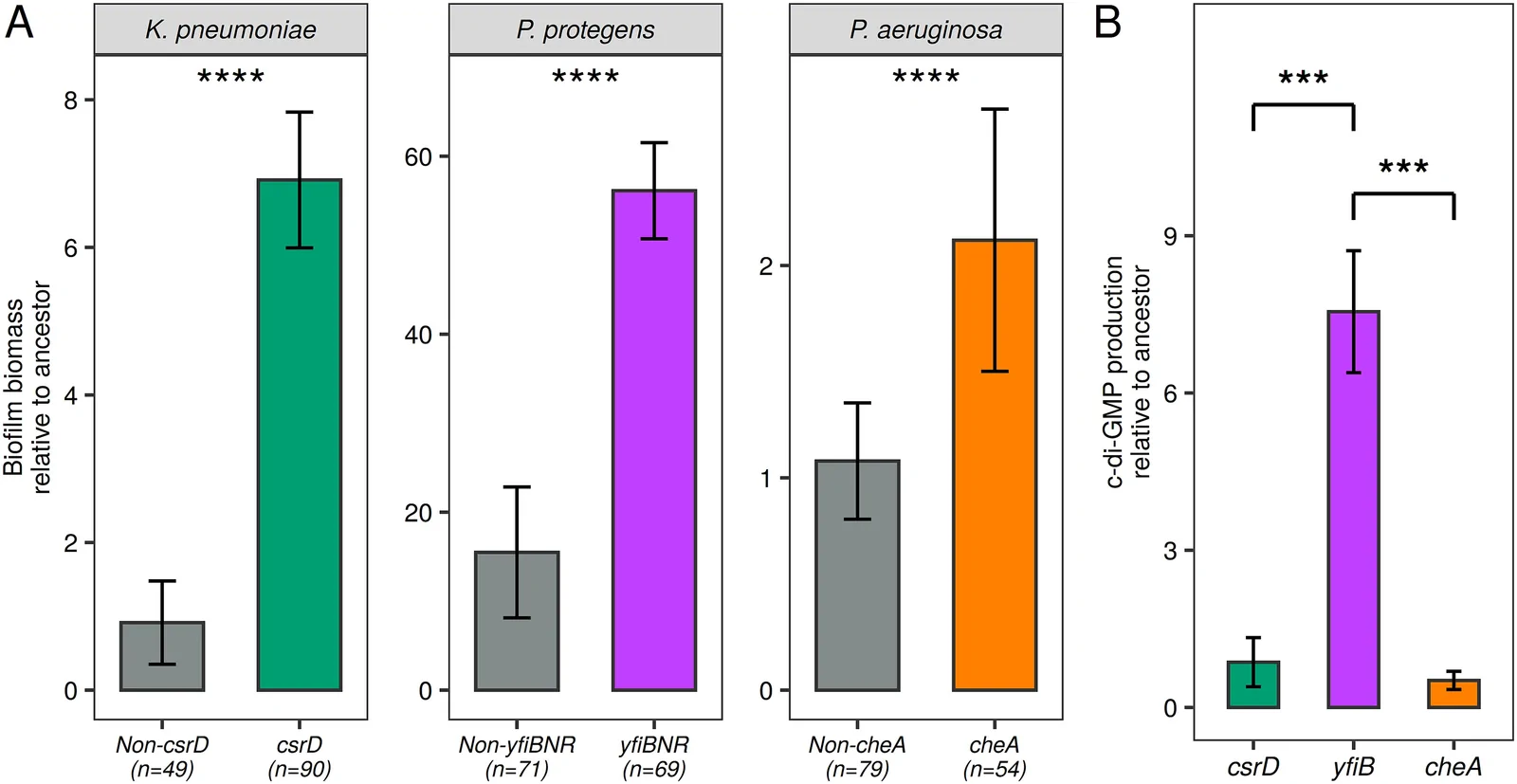
Mutations in *csrD, yfiBNR,* and *cheA* are associated with the enhanced biofilm formation and cyclic-di-GMP production. (A) Biofilm biomass of isolates with and without mutations in the respective key genes for each species. The number of isolates for each of the categories is shown in the legend in brackets. Biofilm biomass was measured from three technical replicates per isolate using crystal violet staining. (B) Cyclic-di-GMP production capacity of the mixed isolates of *K. pneumoniae, P. protegens,* and *P. aeruginosa* with mutations in *csrD* (*n* = 3), *yfiBNR* (*n* = 3) and *cheA* (*n* = 3), respectively. Cyclic-di-GMP were quantified using ELISA kit and expressed in pg/ml. The relative c-di-GMP production capacity compared to the respective ancestral strain is shown. Statistical significance was determined using the Kruskal–Wallis test, followed by pairwise Wilcoxon rank-sum tests with Benjamini–Hochberg correction and significance indicated as: ^*^P < .05; ^**^P < .01; ^***^P < .001; ^****^P < .0001.

Isolates carrying mutations in *csrD, yfiBNR,* or *cheA* showed significantly greater biofilm formation than those lacking such mutations (Fig. 6A), consistent with these mutations being adaptive in the biofilm context, though allelic exchange experiments would be required to establish causation directly. Specifically, *K. pneumoniae* isolates with *csrD* mutations showed an ~7.5-fold increase in biofilm formation relative to non-*csrD* isolates (*P* < .05). *P. protegens* isolates with *yfiBNR* mutations displayed an ~3.5-fold increase (*P* < .05), and *P. aeruginosa* isolates with *cheA* mutations showed an approximately two-fold increase (*P* < 0.05).

Since increased biofilm formation is often associated with elevated intracellular c-di-GMP, we quantified c-di-GMP levels in isolates carrying mutations in *csrD, yfiB*, and *cheA*. The assay revealed that *P. protegens* isolates with mutations in *yfiB* exhibited an approximately eight-fold increase in c-di-GMP levels compared to the ancestral strain (Fig. 6B). In contrast, *K. pneumoniae* and *P. aeruginosa* isolates harboring mutations in *csrD* and *cheA*, respectively, showed no substantial change in c-di-GMP production relative to their ancestral counterparts.

The dominant nSNP identified in the *yfiB* gene resulted in an amino acid substitution (T32P, ACC → CCC) located within the N-terminal region of YfiB (Supplementary Fig. S5) a region previously been implicated in interactions with YfiR. Earlier structural studies have shown that single amino acid substitutions in this domain can promote conformational changes that facilitate sequestration of YfiR into a stable complex, thereby relieving YfiR-mediated repression of YfiN and leading to increased c-di-GMP production [27, 28]. Whether the T32P substitution alters YfiB–YfiR binding in this manner remains to be experimentally tested.

### Mutations in *yfiBNR* are associated with the strongest shift in long-term co-evolutionary dynamics

**Figure 7:**
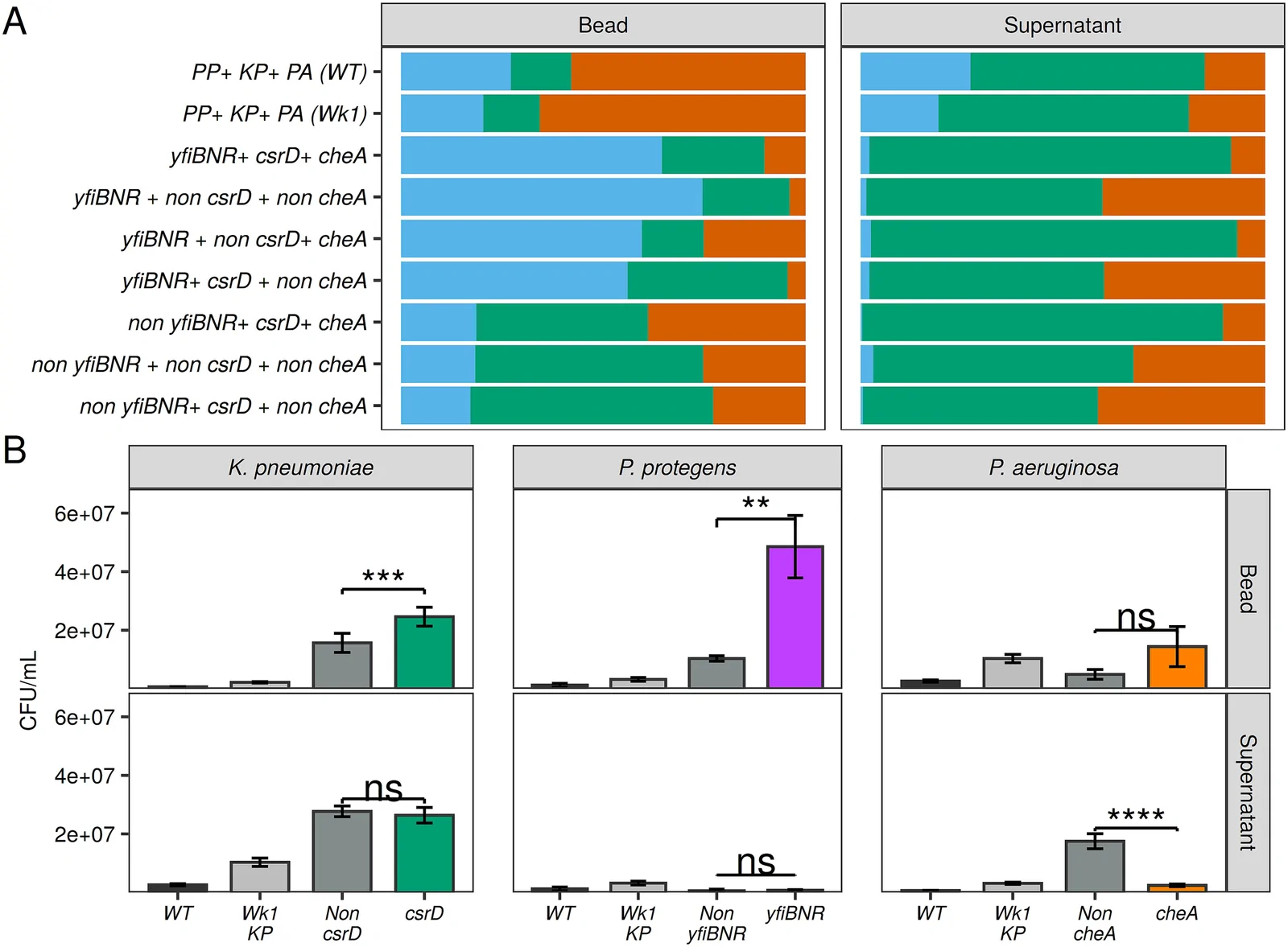
Mutations in *csrD, yfiBNR*, and *cheA* are associated with the observed community dynamics during LTEE. (A) Relative abundance of mixed isolates of *K. pneumoniae, P. protegens*, and *P. aeruginosa* with and without mutations in *csrD, yfiBNR*, and *cheA*, respectively. (B) Differences in cell counts during competition assays between mixed isolates of the three species. Data are presented as means, with error bars indicating 95% confidence intervals. Statistical significance was determined using the Kruskal–Wallis test: ^*^P < .05; ^**^P < .01; ^***^P < .001; ^****^P < .0001.

We further hypothesized that these mutations also influence the relative abundance of the three species within mixed communities, driving the species dynamics observed during the LTEE. To investigate this, we selected mixed isolates of *K. pneumoniae, P. protegens,* and *P. aeruginosa* carrying the most prevalent mutation types in *csrD, yfiBNR,* and *cheA*, respectively, as identified in Supplementary Fig. S4: the nSNP A → G (L313P) in *csrD*, the nSNP A → C (T32P) in *yfiB*, and the 32 bp deletion in *cheA*. Matched nonmutant isolates lacking these focal mutations were selected from the same timepoints (Weeks 8, 15, and 24). The complete mutation profiles of all isolates used in the competition assay, including co-occurring mutations, are provided in Supplementary Table S4. Competition assays were then performed under LTEE-like conditions. Strikingly, mutations in *yfiBNR* were associated with a dominance of *P. protegens* in the biofilm fraction, closely mirroring LTEE community composition (Fig. 7A). In contrast, competitions with ancestral strains or Week-1 isolates resulted in high *P. aeruginosa* abundance in the bead fraction and high *K. pneumoniae* abundance in the supernatant. When *yfiBNR*-mutated isolates were present, *P. protegens* constituted ~60%–80% of the bead-associated population, whereas their absence led to increased bead-associated abundance of *K. pneumoniae*, followed by *P. aeruginosa*. In the supernatant fraction, *K. pneumoniae* dominated regardless of the specific mutant combination.

Quantitative cell counts revealed that *K. pneumoniae* isolates with *csrD* mutations had significantly higher bead-associated abundance compared to non-*csrD* isolates (Fig. 7B). Similarly, *yfiBNR*-mutated *P. protegens* displayed significantly higher bead-attached cells relative to nonmutated counterparts. Planktonic growth curve assays further revealed *csrD* and *cheA* mutants displayed growth rates and yields comparable to the ancestor, whereas *yfiB* mutants of *P. protegens* exhibited reduced μ_max and yield, consistent with a planktonic cost accompanying the biofilm advantage conferred by elevated c-di-GMP (Supplementary Fig. S6).

Although *cheA*-mutated *P. aeruginosa* had slightly higher bead-attached counts than nonmutated isolates, the difference was not statistically significant. In contrast, non-*cheA* isolates had significantly greater planktonic abundance. Growth curve assays showed no meaningful differences in growth rate (~0.31–0.33 h^−1^), lag, or yield between *cheA*, non-*cheA*, and ancestral isolates (Supplementary Fig. S6), indicating that the planktonic advantage of non-*cheA* isolates reflects altered surface attachment or dispersal behaviour rather than intrinsic growth differences.

Collectively, these results reveal associations between *csrD, yfiBNR* and *cheA* mutations, and enhanced biofilm formation and altered populations structure within multispecies communities. Among the three, *yfiBNR* mutations in *P. protegens* exerted the strongest effect, on competitive dominance, consistent with patterns observed during LTEE. This suggests that specific genetic changes are associated with phenotypic adaptation and long-term ecological dynamics in mixed-species biofilms.

## Discussion

Our LTEE of mono- and mixed-species biofilm communities demonstrates that species interactions fundamentally shape the trajectory of microbial adaptation. In monoculture, all three species, *K. pneumoniae, P. protegens*, and *P. aeruginosa*, evolved toward increased biofilm production and phenotypic diversification via distinct genetic pathways. In contrast, mixed-species communities exhibited constrained phenotypic diversity and altered population dynamics, with *P. protegens* emerging as the dominant biofilm resident over 24 weeks. These findings indicate that interspecies interactions can limit the breadth of adaptive evolution while concentrating selection pressure on genetic loci associated with biofilm formation and competitive dynamics.

In monoculture, biofilm biomass increased steadily over time, consistent with selection for traits that enhance attachment and persistence in structured habitats [29, 30]. In mixed-species communities, however, increased biofilm production was consistently lower across all species than in monoculture, suggesting that interspecies interactions constrain this trait, consistent with competitive and antagonistic interactions previously documented in this three-species model [6, 8, 18]. Prior short-term characterisation of this same community demonstrated enhanced stress resistance, interspecies functional diversity, and coexistence modulated by AI-2 transporters, quorum sensing, and associational resistance to predation [6, 8, 17–20], with no single species stably dominating over 24–72 h. Our LTEE reveals this equilibrium to be evolutionarily transient over ~400 generations, *P. protegens* progressively displaced both *K. pneumoniae* and *P. aeruginosa* from bead-associated biofilms, coinciding with the emergence of mutations in the *yfiBNR* operon, a central regulator of cyclic-di-GMP signaling and biofilm formation in *Pseudomonas* sp. [26, 31]. Its dominance in both beads and supernatants from Week 10 onward reflects a combination of biofilm advantage and planktonic competitiveness, with growth curve assays pointing to niche partitioning within the population. The *yfiBNR* mutants are associated with biofilm dominance at a cost to planktonic growth, while non-*yfiB* genotypes sustain competitive fitness in the supernatant.

Whole-genome sequencing revealed that specific mutations in *csrD, yfiBNR,* and *cheA* arose early and persisted across the LTEE. Competition assays showed that these mutations were associated with enhanced biofilm formation, with *yfiBNR* variants in *P. protegens* exerting the largest effect on competitive dominance. The *csrD* mutations likely impact the turnover of regulatory RNAs controlling glycogen storage and biofilm-related pathways, while *cheA* indels in *P. aeruginosa* may alter chemotaxis responses, shifting the balance between surface colonization and dispersal [32]. Mutations targeting global regulatory networks and cyclic-di-GMP signaling pathways are a recurrent outcome of biofilm-focused experimental evolution [33, 34], and our findings align closely with this pattern. The ecological effects of these mutations were context-dependent, *cheA* mutations increased biofilm formation in *P. aeruginosa* but did not confer dominance in the bead fraction when competing against *yfiBNR*-mutated *P. protegens*. This highlights that the fitness effect of a given mutation cannot be inferred from monoculture performance alone and depends on the identity and evolutionary state of competing species [35]. A limitation of the present study is that the genotype–phenotype links reported here remain correlational. Allelic exchange of *csrD, yfiBNR*, and *cheA* mutations into ancestral backgrounds, alongside structural and binding assays of the YfiB–YfiR interaction, will be required to establish causation definitively.

Phenotypic profiling and PCA demonstrated that monoculture evolution yielded greater trait diversity than co-evolution, particularly in bead-associated populations. This aligns with theory predicting that strong competitors in fluctuating environments can favor a narrower set of successful strategies, thereby reducing diversification [36, 37]. The constraint on phenotypic diversity in mixed-species populations likely reflects both ecological filtering, where only certain phenotypes persist under competitive pressure, and a narrowed fitness landscape imposed by persistent interspecies interactions. Notably, such constraints did not preclude community stability, as evidenced by stable bead-to-supernatant ratios from mid-experiment onwards. Per-species bead-associated abundance also differed between conditions, with *P. aeruginosa* and *K. pneumoniae* reaching lower densities in mixed communities and thus potentially fewer realized generations; however, *P. protegens* reached comparable abundance in both contexts yet still showed reduced diversification, indicating that generation number alone cannot fully explain the constrained pattern.

Our findings have direct implications for understanding microbial community assembly in natural biofilms and engineered systems. Keystone mutations such as those in *yfiBNR* can rapidly reshape community structure, potentially overriding the effects of initial community composition, consistent with observations from clinical and environmental biofilms [38, 39]. From an applied perspective, targeting specific regulatory pathways could selectively disrupt dominant biofilm formers in polymicrobial infections or industrial contexts.

Overall, this work demonstrates that microbial evolution in multispecies environments cannot be predicted from single-species trajectories alone. Species interactions both constrain and direct adaptation, channeling evolution towards a limited set of ecological strategies driven by a small number of high-impact mutations.

## Supplementary Material

Mixed_species_biofilm_manuscript_Supplementary_file_revised_ycag237

## Data Availability

Sequence data generated in this study are available from the NCBI Sequence Read Archive with accession number PRJNA1401427.
