# Supplementary material for: Mixed-species interactions constrain diversification and shape biofilm evolution
Source: ISME Commun. 2026 Aug 20;6(1):ycag237. doi: 10.1093/ismeco/ycag237 (PMC13584209; doi:10.1093/ismeco/ycag237)
Supplement: Supplementary file 1 [file mixed_species_biofilm_manuscript_supplementary_file_revised_ycag237.docx]

# Supplementary Information

Mixed-species interactions constrain diversification and shape biofilm evolution

Sanaa Harrass^1^, Joyce To^1^, Parisa Noorian^1^, Gustavo Espinoza-Vergara^1^, Vaughn S. Cooper^2^, Scott A. Rice^1^, Diane McDougald^1^, M. Mozammel Hoque^1,3,*^

^1^Australian Institute for Microbiology & Infection, University of Technology Sydney, Sydney, NSW 2007, Australia.

^2^Center for Evolutionary Biology and Medicine, University of Pittsburgh, School of Medicine, Pittsburgh, PA, USA.

^3^School of Engineering, Faculty of Engineering and Information Sciences, University of Wollongong, NSW 2500, Australia.

*Correspondence to: mdmozammel.hoque@uts.edu.au, mhoque@uow.edu.au,

Running Title: Long-term evolution of a mixed species biofilms.

**Keywords:** Biofilm, LTEE, diversity, evolution, phenotypes, mutation.

**Content:**

Supplementary Table Text

Supplementary Table S1-S4

Supplementary Figures S1-S6

**Supplementary Text**

## Genetic signatures of adaptation reveal species-specific evolutionary trajectories

***Mutation accumulation.*** The distribution of mutations per isolate varied across species. *K. pneumoniae* isolates carried an average of 1.8 mutations (range: 0–7), *P. protegens* isolates averaged 4.6 mutations (range: 0–17), and *P. aeruginosa* isolates averaged 9.2 mutations (range: 5–12). In *K. pneumoniae*, both community context and time significantly predicted mutation accumulation across both bead and supernatant fractions (two-way ANOVA, community: F = 30.7, P < 0.001; time: F = 46.6, P < 0.001), without a significant interaction (P = 0.097) (Fig. 4A and Supplementary Table S3). By contrast, *P. protegens* showed no significant community effect in either fraction (combined: F = 1.7, P = 0.18), with mutation accumulation increasing significantly with time in the supernatant fraction only (time: F = 7.07, P < 0.001). In *P. aeruginosa*, a significant community effect was detected in the bead fraction (F = 5.20, P = 0.025), though this was not accompanied by a significant interaction with time (P = 0.1), and no community effect was observed in the supernatant fraction (P = 0.3). Mutation accumulation increased significantly with time in both fractions for *P. aeruginosa* (both P < 0.001).

***Unique and shared mutated genes.*** In *P. aeruginosa*, more genes were mutated in isolates from mono-species populations than from multispecies communities: 56% (n = 23) of mutated genes were unique to mono isolates, 27% (n = 11) were unique to mixed isolates, and 17% (n = 7) were shared (Fig. 4B). Conversely, in *P. protegens*, fewer genes were mutated in mono-species isolates than in mixed-species isolates: 13% (n = 5) were unique to mono isolates, 68% (n = 26) were unique to mixed isolates, and 18% (n = 7) were shared. Unlike the *Pseudomonas* species, *K. pneumoniae* showed comparable proportions of unique mutations in mono (48%, n = 20) and mixed (40%, n = 17) isolates, with 12% (n = 5) of mutated genes shared between them.

***Mutation types.*** Analysis of mutation types revealed a higher proportion of small indels from week 8 onward in mixed species bead isolates of *K. pneumoniae* and *P. protegens*, and in *K. pneumoniae* supernatant isolates (Supplementary Fig. S2). In *P. aeruginosa*, small indels were the predominant mutation type overall with no clear temporal trend. Notably, *P. aeruginosa* accumulated fewer mutations in mixed than mono communities while *P. protegens* showed the opposite pattern; these differences partially reflect the population dynamics in Fig. 1, where *P. aeruginosa* declined sharply in mixed communities while *P. protegens* became dominant, influencing isolate representation at later sampling timepoints.

Parallel evolution was defined by distinct mutations in the same gene arising over time within a replicate population and independently across separate replicate populations.

***csrD in K. pneumoniae****.* Mutations in *csrD* (carbon storage regulator/RNase E specificity factor) emerged by week 8 and persisted through week 24 (Fig. 5). Both non-synonymous mutations and small indels were detected across independent replicate populations, together accounting for 60–90% of sequenced mixed isolates from weeks 8 to 24. Notably, multiple distinct *csrD* mutations arose in parallel across different genetic backgrounds within and between replicate populations, a pattern consistent with a soft selective sweep on *csrD* function. The comparable frequency of *csrD* mutations in bead-associated and planktonic fractions most likely reflects shared ancestry through dispersal from the colonized bead at each transfer rather than independent parallel selection in both niches, though this pattern is consistent with *csrD* mutations not incurring a strong fitness cost in the planktonic phase. Whole-population sequencing confirmed a steady increase in *csrD* mutation frequency over time, reaching 25%, 50%, and 75% at weeks 8, 15, and 24, respectively (Supplementary Fig. S3). Although *csrD* mutations also occurred in mono isolates, they were less frequent and temporally restricted (e.g., weeks 8 and 24 in bead-associated isolates, week 8 only in supernatant isolates). An in-depth analysis identified a dominant nSNP at position 938 (A->G) in *csrD* (Supplementary Fig. S4).

***yfiBNR in P. protegens****.* Mutations were observed in the *yfiBNR* operon (*yfiB*, *yfiN*, *yfiR*) from weeks 8 to 24, with this operon showing parallel mutations across 3 timepoints in mixed communities. YfiBNR is a tripartite signaling module that controls intracellular c-di-GMP levels and small colony variant formation. Notably, *yfiB* mutations occurred exclusively in mixed isolates, affecting ~20% of bead-associated isolates, whereas *yfiN* and *yfiR* mutations were present in both mono and mixed isolates. Whole-population data mirrored these patterns, with *yfiB* mutated only in mixed populations, while *yfiN* and *yfiR* mutations were distributed across both evolutionary contexts (Supplementary Fig. S3). The most prevalent mutation among mixed *P. protegens* isolates was a nSNP at position 94 (A->C) in *yfiB*, resulting in a threonine-to-proline substitution (T32P) in the periplasmic domain. This residue lies within a region critical for YfiB's interaction with YfiR, suggesting that the mutation may disrupt sequestration of YfiR and consequently elevate c-di-GMP levels to promote biofilm formation.

***cheA and c-di-GMP loci in P. aeruginosa.*** Small indel mutations in the chemotaxis gene cheA were unique to mixed isolates and persisted from week 8 to week 24. At week 8, 30% of bead-associated and 10% of planktonic isolates carried *cheA* mutations; by week 15, these proportions rose to 100% and 60%, respectively, before declining slightly at week 24. Conversely, *dgcN* (PA3177), encoding a diguanylate cyclase, was mutated only in mono isolates, with frequencies increasing over time across 3 timepoints. In bead-associated mono isolates, *dgcN* mutation prevalence rose from 30% (week 8) to 60% (week 15) and 80% (week 24); a similar upward trend was observed in planktonic isolates (30%, 50%, and 60% over the same intervals). In addition to *dgcN*, mutations in PA0861 (*rbdA*) were detected in mono isolates (Supplementary Fig. S3). Unlike DgcN, RbdA functions as a phosphodiesterase that degrades c-di-GMP and promotes biofilm dispersal. The occurrence of mutations in both c-di-GMP synthesis (*dgcN*) and degradation (*rbdA*) pathways exclusively in monoculture isolates is noteworthy, though the functional consequences of these mutations for c-di-GMP homeostasis and biofilm regulation remain to be experimentally determined. Whole-population sequencing confirmed an increasing frequency of nSNPs in *dgcN* across mono populations.

**Supplementary Tables**

## Supplementary Table S1: Number of isolates phenotyped across different condition.

## Supplementary Table S2: Phenotypic dispersion test PERMDISP (betadisper).

Homogeneity of multivariate dispersions tested using vegan::betadisper with 999 permutations on PC1+PC2 scores. Mean distance to centroid shown for each group.

## Supplementary Table S3: Two-way ANOVA results for mutation counts.

Model: log(mutations + 1) ~ Community × Time.

| **Species** | **Fraction** | **Community (Mono vs Mixed)** | | | **Time (Week)** | | | **Community × Time** | | |
| --- | --- | --- | --- | --- | --- | --- | --- | --- | --- | --- |
|  |  | **F** | **p** | **Sig.** | **F** | **p** | **Sig.** | **F** | **p** | **Sig.** |
| ***K. pneumoniae*** | Combined | 30.77 | <0.001 | *** | 46.65 | <0.001 | *** | 2.14 | 0.097 | ns |
| ***K. pneumoniae*** | Bead | 16.50 | <0.001 | *** | 21.80 | <0.001 | *** | 1.65 | 0.182 | ns |
| ***K. pneumoniae*** | Supernatant | 15.17 | <0.001 | *** | 26.04 | <0.001 | *** | 1.75 | 0.164 | ns |
| ***P. protegens*** | Combined | 1.78 | 0.184 | ns | 6.38 | <0.001 | *** | 0.51 | 0.678 | ns |
| ***P. protegens*** | Bead | 0.32 | 0.573 | ns | 1.05 | 0.375 | ns | 1.05 | 0.375 | ns |
| ***P. protegens*** | Supernatant | 1.82 | 0.181 | ns | 7.07 | <0.001 | *** | 0.81 | 0.490 | ns |
| ***P. aeruginosa*** | Combined | 5.09 | 0.025 | * | 14.18 | <0.001 | *** | 1.45 | 0.231 | ns |
| ***P. aeruginosa*** | Bead | 5.20 | 0.025 | * | 7.18 | <0.001 | *** | 2.10 | 0.107 | ns |
| ***P. aeruginosa*** | Supernatant | 0.97 | 0.327 | ns | 6.86 | <0.001 | *** | 0.52 | 0.667 | ns |

## Supplementary Table S4. Mutations present in isolates from mixed communities used in the competition assay.

**Supplementary Figures**

## **Supplementary Fig. S1: Phenotypes of *Pseudomonas* spp.**

Data are presented as the mean of three technical replicates of respective mono and mixed isolates as described in Supplementary Table 1. The error bar represents 95% CI. Statistical significance was determined using Kruskal-Wallis test between mono and mixed isolates and indicated as *, P < 0.05; **, P < 0.01; ***, P < 0.001 and ****, P < 0.0001.

## Supplementary Fig. S2: Mutation profiles of mono and mixed isolates.

This stacked bar chart displays the relative frequency of different mutation types, with each color representing a specific mutation type as defined in the legend. A red cross indicates insufficient sequencing reads for the corresponding species and week, leading to no data analysis for that point.

## Supplementary Fig. S3: Mutational frequency in key genes from whole population sequencing data.

×

×

×

×

×

×

×

This plot highlights the frequency of mutations (including nSNPs and indels) in specific genes that appeared in week 8 and persisted through weeks 15 or 24. A red cross indicates insufficient sequencing reads for the corresponding species and week, leading to no data analysis for that point. Grey dots represent the frequency trajectories of all other genes carrying nSNPs or indels shown as background reference.

## Supplementary Fig. S4: Mutational spectrum in key genes of *K. pneumoniae*, *P. protegens* and *P. aeruginosa*.

This heatmap illustrates the spectrum of mutations (nSNPs and indels) in key genes. The color and numerical values within the heatmap represent the fraction of isolates carrying a mutation in the corresponding gene.

## **Supplementary Fig. S5.** Alphafold predicted protein structural model of CsrD, YfiB and CheA highlighting the mutations of interest in pink.

## Supplementary Fig. S6. Planktonic growth curves. The growth curves were performed for ancestors (WT), *csrD*-mutated, non-*csrD*, *yfiB*-mutated, non-*yfiB*, *cheA*-mutated, and non-*cheA* isolates (Week 24, Mixed communities) in MCG medium. Overnight cultures were normalised to OD₆₀₀ = 0.05 and transferred to a 96-well plate. OD₆₀₀ was measured every 10 minutes over 16 hours at 24°C using an Eppendorf 2200 plate reader. Three technical replicates were performed per strain. Maximum growth rate (µ_max), lag time, and final yield were extracted from the resulting curves.
